# Supplementary material for: Strong transient magnetic fields induced by THz-driven plasmons in graphene disks
Source: Nat Commun. 2023 Nov 18;14:7493. doi: 10.1038/s41467-023-43412-x (PMC10657377; doi:10.1038/s41467-023-43412-x)
Supplement: Supplementary file 1 — Supplementary information [file 41467_2023_43412_MOESM1_ESM.pdf]

Supplementary information

Strong transient magnetic fields induced by THz driven plasmons in graphene disks

Jeong Woo Han[1], Pavlo Sai[2], Dmytro But[2], Ece Uykur[3], Stephan Winnerl[3], Gagan Kumar[4], Matthew L. Chin[5], Rachael L. Myers-Ward[6], Matthew T. Dejarld[6], Kevin M. Daniels[5], Thomas E. Murphy[5], Wojciech Knap[2] and Martin Mittendorff[1,*]

[1]Universität Duisburg-Essen, Fakultät für Physik, 47057 Duisburg, Germany
[2]CENTERA Laboratories, Institute of High Pressure Physics PAS, 01-142 Warsaw, Poland,
[3]Helmholtz-Zentrum Dresden-Rossendorf, Dresden 01328, Germany
[4]Indian Institute of Technology, Guwahati, Assam 781039 India
[5]University of Maryland, College Park, MD 20740 Maryland, USA
[6]U.S. Naval Research Laboratory, Washington, DC 20375, USA
**Corresponding author: martin.mittendorff@uni-due.de*

Section S1. Sample characterization

Section S2. Derivation of optical conductivity tensor

Section S3. Interpretation of the sign crossover of Faraday rotation

Section S4. Circularity of pump beam

Section S5. Temperature dependent pump beam induced Faraday rotation

Section S6. Change of polarization of probe beam by linearly polarized pump beam

Section S7. Extraction of θ_F

Section S1. Sample characterization

In order to characterize our sample, i.e., graphene disks, we measured the transmission spectrum at 10 K using Fourier-transform infrared spectrometer. Figure S1 (a) shows the transmission spectrum of graphene disks. We confirmed that the plasmon resonance frequency ω_p is located at 3.5 THz. To analyze further, we fitted the transmission spectrum using equivalent sheet conductivity σ, given by[1,2]:

$$\sigma = f\cdot\frac{D}{\pi\left(\Gamma - \frac{i\left(\omega^2-\omega_p^2\right)}{\omega}\right)} \tag{S1}$$

where f, D, and Γ are the filling factor, Drude weight, and scattering rate, respectively. The extracted parameters from the transmission spectrum (D and Γ) and related values calculated from extracted parameters are summarized in Table S1.

Symbol	Name	Value
R	Disk radius	0.6 μm
Λ	Disk Periodicity	1.5 μm
$f=\pi R^2/\Lambda^2$	Filling factor	0.5
D	Drude weight	$1.2\cdot10^{11}\ \Omega^{-1}s^{-1}$
v_F	Fermi velocity	10^8 cm s^{-1}
e	Elementary charge	$1.6\cdot10^{-19}$ C
$n=(D\hbar/e^2v_F)^2/\pi$	Carrier concentration	$7.83\cdot10^{12}$ cm^{-2}
Γ	Scattering rate	$9.31\cdot10^{12}$ s^{-1}
ω_p	Plasmon frequency	3.5 THz

Table. S1: Extracted parameters from the fitting result on the transmission spectrum.

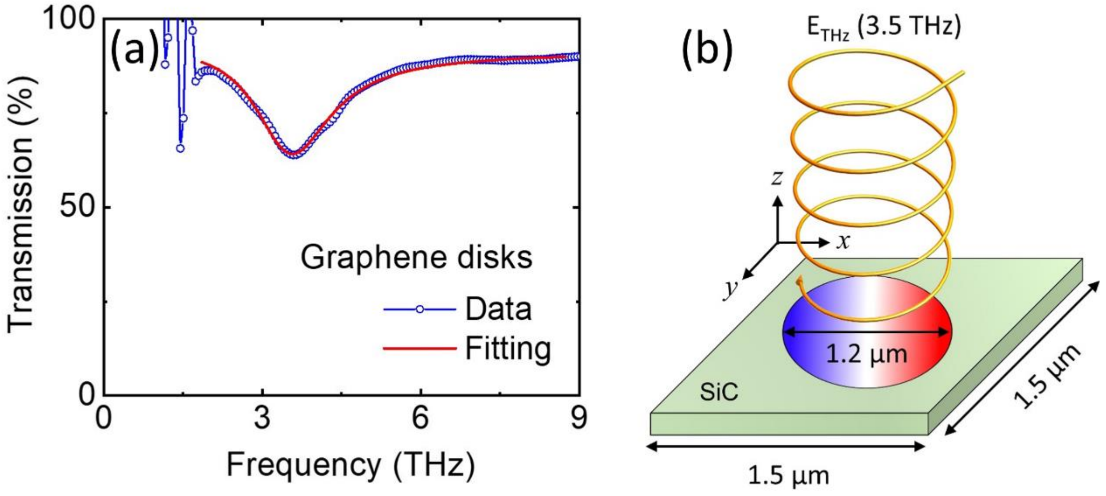

Fig. S1: (a) Transmission spectrum of graphene disks. Symbols and line show the experimental data and the fitting result, respectively. (b) Geometry of the graphene disk as used for the COMSOL simulations.

Section S2. Derivation of optical conductivity tensor

The optical conductivity tensor σ for a graphene disk array can be obtained by using a simple harmonic oscillator in a magnetic field[3], which gives coupled differential equations:

$$\ddot{x}+\Gamma\dot{x}+\omega_p^2 x=eE_0e^{i\omega t}+\omega_c\dot{y} \tag{S2}$$

$$\ddot{y}+\Gamma\dot{y}+\omega_p^2 y=-\omega_c\dot{x} \tag{S3}$$

where ω_p, Γ, ω_c, and E_0 are the plasmon frequency at zero magnetic field, scattering rate, cyclotron frequency, and the strength of applied electric field. As general solutions for Eq. S2 and S3, we set as:

$$x=A1*\frac{1}{\Gamma-i\frac{\omega^2-\omega_c\omega-\omega_p^2}{\omega}}e^{-i\omega t} \tag{S4}$$

$$y=A2*\frac{1}{\Gamma-i\frac{\omega^2-\omega_c\omega-\omega_p^2}{\omega}}e^{-i\omega t} \tag{S5}$$

By inserting Eq. (S4)-(S5) into (S2) and (S3), the coefficients A1 and A2 can be obtained. The full expressions of x and y are given by:

$$x=-\frac{eE_0(-i\omega^2+i\omega_p^2+\omega\Gamma)}{m_c\omega(\omega^2-\omega_p^2+\omega\omega_c+i\omega\Gamma)}*\frac{1}{\Gamma-i\frac{\omega^2-\omega_c\omega-\omega_p^2}{\omega}}e^{-i\omega t} \tag{S6}$$

$$y=\frac{eE_0\omega_c}{m_c\omega(\omega^2-\omega_p^2+\omega\omega_c+i\omega\Gamma)}*\frac{1}{\Gamma-i\frac{\omega^2-\omega_c\omega-\omega_p^2}{\omega}}e^{-i\omega t} \tag{S7}$$

The electric current J is defined as J=*nev* where *n*, *e*, and *v* are the carrier density, electric charge, and the drift velocity, respectively. And each component of *v* for *x* and *y* directions can be obtained by differentiation with respect to time t which are:

$$\frac{dx}{dt}=v_x=\frac{ie\omega(\omega^2-\omega_p^2-i\omega\Gamma)}{m_c(\omega^2-\omega_p^2+\omega\omega_c+i\omega\Gamma)(\omega^2-\omega_p^2-\omega\omega_c+i\omega\Gamma)}\mathbf{E} \tag{S8}$$

$$\frac{dy}{dt}=v_y=\frac{e\omega^2\omega_c}{m_c(\omega^2-\omega_p^2+\omega\omega_c+i\omega\Gamma)(\omega^2-\omega_p^2-\omega\omega_c+i\omega\Gamma)}\mathbf{E} \tag{S9}$$

where $\mathbf{E}$ is the unit electric field of $\mathbf{E}=E_0 \cdot e^{-i\omega t}$. In order to obtain σ_{xy}, we used the relation between the electric current and the applied electric field:

$$\begin{pmatrix} J_x \\ J_y \end{pmatrix}=\frac{1}{\sqrt{2}}\begin{pmatrix} \sigma_{xx} & \sigma_{xy} \\ -\sigma_{yx} & \sigma_{yy} \end{pmatrix}\begin{pmatrix} \mathbf{E}_x \\ \mathbf{E}_y \end{pmatrix} \tag{S10}$$

We only apply the electric field in the *x*-direction thus $\mathbf{E}_y=0$. Thus, we can obtain the relations of $J_x=\sigma_{xx}\mathbf{E}_x/\sqrt{2}$ and $J_y=-\sigma_{yx}\mathbf{E}_x/\sqrt{2}$. From these relations, we can finally obtain σ_{xx} and σ_{xy} as:

$$\sigma_{xx}=\frac{fD}{\sqrt{2}}\frac{i\omega(\omega^2-\omega_p^2-i\omega\Gamma)}{(\omega^2-\omega_p^2+\omega\omega_c+i\omega\Gamma)(\omega^2-\omega_p^2-\omega\omega_c+i\omega\Gamma)} \tag{S11}$$

$$\sigma_{xy}=\frac{fD}{\sqrt{2}}\frac{\omega^2\omega_c}{(\omega^2-\omega_p^2+\omega\omega_c+i\omega\Gamma)(\omega^2-\omega_p^2-\omega\omega_c+i\omega\Gamma)} \tag{S12}$$

Here, *D* is the Drude weight. Since graphene disks are array structures, we inserted the filling factor *f* to compensate for the effective area of graphene.

Section S3. Interpretation of the sign crossover of Faraday rotation

In this section, we discuss the sign crossover of Faraday rotation θ_F as a function of frequency. For example, the sign of θ_F is changed from positive to negative with the onset frequency of around 1.5 THz in the case of unpatterned graphene with the magnetic field being 1 T (see Fig. 1**d** in main manuscript). We interpret that this crossover is related to the phase delay between the driving electric field and the motion of electrons. In more detail, no phase delay occurs at low frequency, close to the direct current regime, whereas the phase delay of the charge carrier motion increases with frequency and eventually the direction of electron displacement becomes phase shifted by 180° with respect to the driving electric field. To quantify this effect, we considered the complex Drude optical conductivity of graphene σ under the magnetic field enviorment[4].

$$\sigma = \frac{e^2 \varepsilon_F}{\hbar^2 \pi} \cdot \frac{i(\omega + i\Gamma)}{(\omega + i\Gamma)^2 - \omega_c^2} \tag{S13}$$

We used parameters in Eq. S13 from Table S1. The complex magnetic-field-dependent Drude optical conductivity is provided in Fig. S2(a) and (b). In order to consider the absorption and reemission process, we considered the dipole radiation process. In this radiation process, the phase of the position of charge-carriers plays a crucial role. The relative phase information φ between the incident light and the position of carriers can be extracted from Eq. S13 and it is given by:

$$\varphi = \tan^{-1}\left(-\Gamma \cdot \frac{\omega}{\omega_c^2 - \omega^2}\right) \tag{S14}$$

The extracted phases depending on the magnetic field are shown in Fig. S2(c). As can be seen, the phase shift is zero for lowest frequencies, contrasting to AC region where the imaginary part of σ has non-zero values. And, one can observe that the phase exceeds 90 degrees upon increasing the frequency, suggesting that the oscillating displacement becomes opposite compared to the incoming light wave.

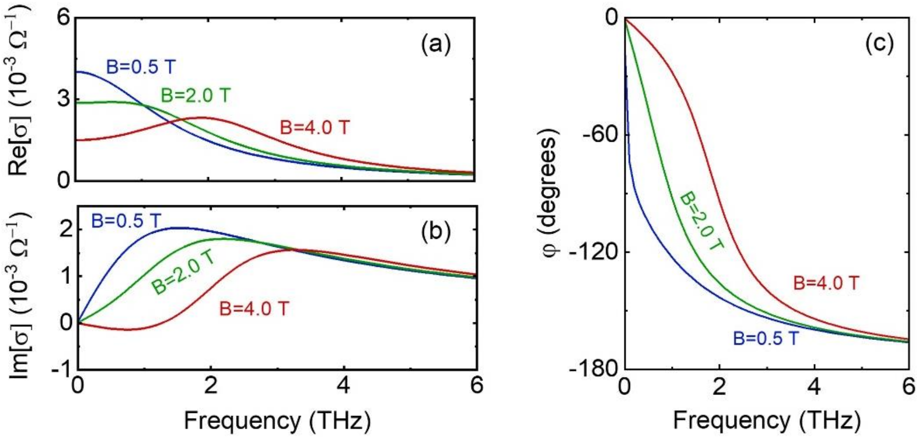

Fig. S2: (a) Real and (b) imaginary part of complex Drude optical conductivity of graphene σ under the magnetic field. (c) Relative phase difference φ between the incident light the position of charged carriers.

Considering the extracted phase between electron displacement and incident electric field E, we computed the trajectories of electrons under the magnetic fields B by solving coupled equations, as:

$$\frac{d^2 y}{dt^2} = \frac{eB}{m^*}\frac{dz}{dt} = \omega_c \frac{dz}{dt} \tag{S15}$$

$$\frac{d^2 z}{dt^2} = \frac{eE}{m^*} - \frac{eB}{m^*}\frac{dy}{dt} = \omega_c\left(\frac{E}{B} - \frac{dy}{dt}\right) \tag{S16}$$

where m^* is the effective mass of electrons. The Electric E and magnetic B fields are set to be applied to z and x directions, respectively (See. Fig. S3(a)).

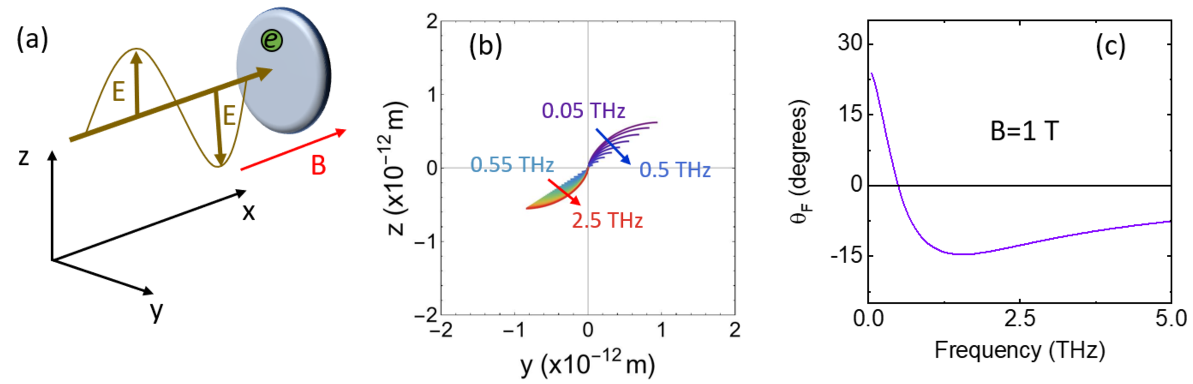

Fig. S3: (a) Geometry of calculation. (b) Trajectories of electrons. Frequency is varied from 0.05 THz to 2.5 THz (c) Extracted Faraday rotation the trajectories of electrons.

Figure S3 (b) shows the calculated trajectories of electrons after 1 ps. For this calculation, we set strengths of electric and magnetic fields as 1 V m^{-1} and 1 T, respectively. Note that the corresponding ω_c to 1 T is about 0.5 THz for this calculation. One can observe that the trajectories of electrons become opposite based on around 0.5 THz. It implies that the phase difference between the incoming electric field and the motion of electrons plays a crucial role in the determination of the trajectories. From obtained trajectories, we extracted θ_F, provided in Fig. S3(c). For the direct comparison to experimental data shown in Fig. 1**d** (main manuscript), we adjust the amplitude of θ_F using the amplitude of complex optical conductivity (Eq. S13) as the scale factor which allows us to consider the absorption of the incident light. Altogether these observations allow us to conclude that the sign crossover originates from the phase shift between the electric fields and the motion of electrons on graphene. Note that this simplistic approach is only meant to help interpreting the change in sign and is not a complete description of the Faraday rotation.

Section S4. Circularity of pump beam

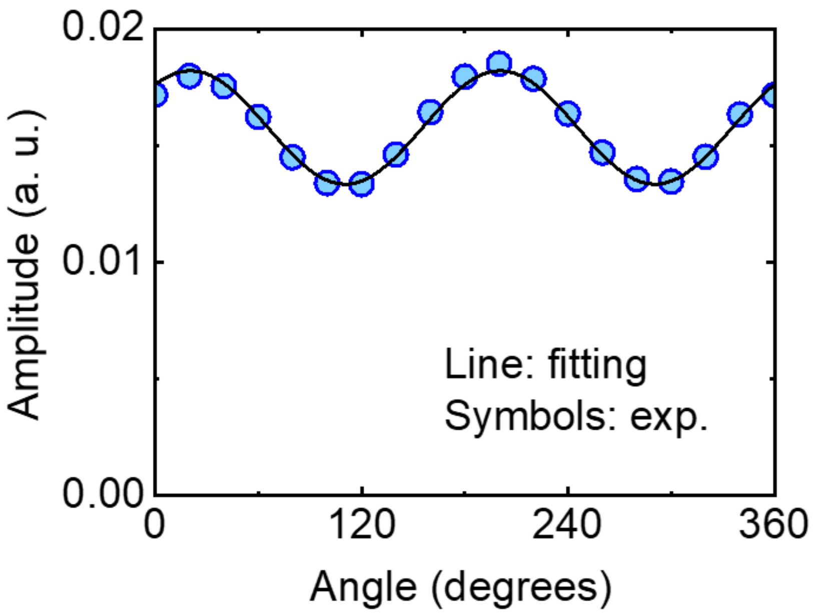

Fig. S4: Amplitude change of circularly polarized pump beam as a function of the rotation angle for wire-grid polarizer.

We observed the sinusoidal oscillation upon varying the angle of wire-grid polarizer, suggesting that our FEL pump beam passing through λ/4 plate is not perfectly circular. In order to quantify the circularity, we fit our experimental data with sinusoidal function of:

$$\text{Amplitude}=A_0\sin(2\vartheta+\varphi)+B_0 \tag{S17}$$

where A_0, ϑ, φ, and B_0 are the coefficient of sine function, the angle of wire-grid polarizer, phase shift, and offset value. From the fitting, these parameters turn out A_0=0.0024, φ =49°, and B_0=0.016. The information of circularity can be obtained by dividing B_0 from A_0, manifesting the fact that our circularly polarized pump beam contains 15 % of the linearly-polarized component.

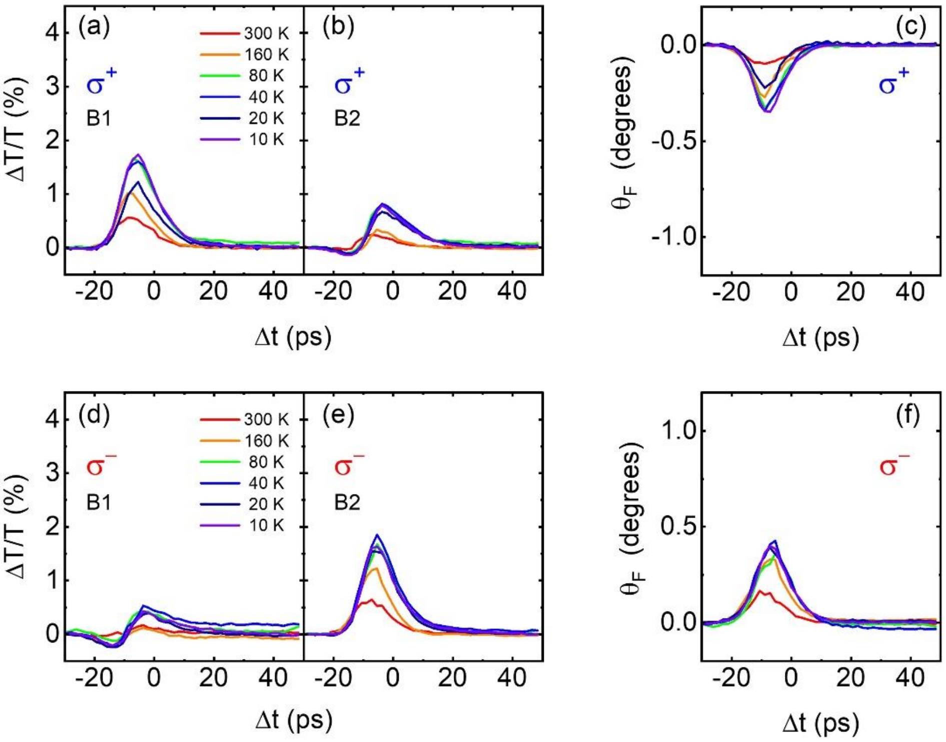

Fig. S5: Circularly polarized pump beam induced transmission change ΔT/T measured at bolometers of B1 (a) and B2 (b). Pump-beam is the left-handed circular polarization σ^+ (d), (e) Corresponding data set to (a) and (b) with the right-handed circular polarization σ^-. (c), (f) Extracted θ_F's from σ^+ and σ^-, respectively.

Section S5. Temperature dependent pump beam induced Faraday rotation

Figure S5 shows the circularly-pump induced Faraday rotation θ_F as a function of temperature. Temperature was varied from 10 K to 300 K. All notation in Fig. S5 is the same as the main manuscript. One can confirm that θ_F is still observable even at room temperature. Input fluence was set to 110 nJ cm^{-2}.

Section S6. Change of polarization of probe beam by linearly polarized pump beam

To exclude that the observed θ_F originates from a pump-induced anisotropy caused by linearly polarized radiation (plasmonic nonlinearity)[5], but the circular motion of the plasmonic current, we investigated the change of the polarization of the probe beam θ_p induced by the linearly polarized pump beam. Figures S6(a) and (b) show the linearly polarized pump beam induced transmission change ΔT/T as a function of the time delay Δt between the pump and the probe pulses. ΔT/T measured at the bolometer B2 exhibits larger changes than the corresponding data set measured at B1. This result implies that the polarization of the probe beam θ_p is rotated by the linearly polarized pump beam (see Fig. S6(c)). At the maximum fluence (420 nJ cm^{-2}), θ_p is confirmed to be changed by 0.5 degrees. Considering the fact that our circularly polarized pump beam contains 15 % of the linear component, we can deduce 15 % of 0.5 degrees (0.075 degrees) could be caused by the linear component of the circularly polarized pump pulse. Thus, we can safely conclude that the plasmonic nonlinearity plays a negligible role in our experiment as the observed θ_F is more than ten times larger compared to θ_p at a comparable pump fluence.

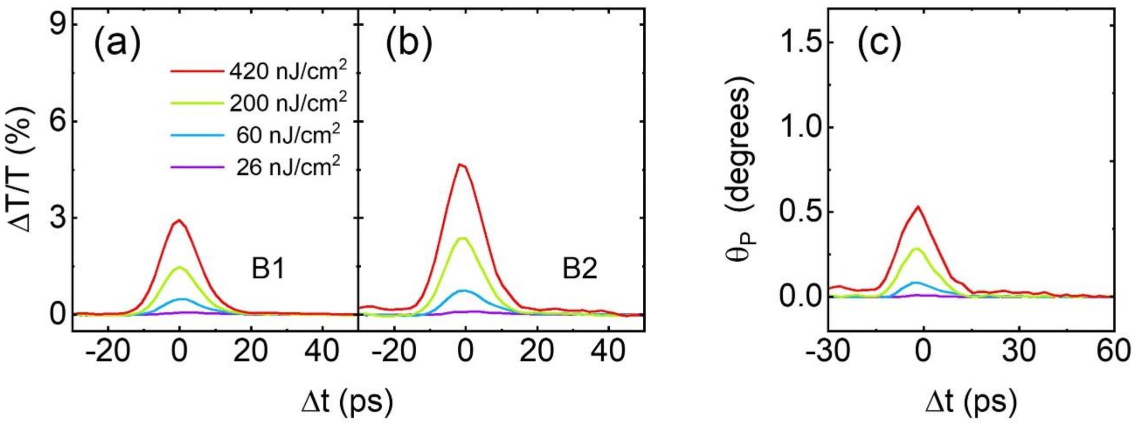

Fig. S6: linearly polarized pump beam induced transmission change ΔT/T measured at bolometers of B1 (a) and B2 (b). (c) Extracted changed polarization of probe beam θ_F.

Section S7. Extraction of θ_F

The wire grid polarizer used to separate the two linear polarization components is set to 45°, i.e. both orthogonal contributions have the same power. The transmission through the polarizer as a function of the polarization angle is in general described by the Cos^2 between the electric field and the direction of the polarizer. When the polarization of the probe beam changes, the power for one bolometer increases

following the Cos^2-law, while the second one decreases accordingly. Thus, the difference between the relative changes of both channels has to be divided by two. Equation S18 shows the equation exploited to determine the Faraday angle. Figure S7 shows the relation between θ_F and $\Delta T/T$.

$$\mathrm{Cos}^2(\theta_F+45^\mathrm{o})=\frac{1}{2}\left(\frac{\Delta T}{T}\bigg|_{\sigma^+}-\frac{\Delta T}{T}\bigg|_{\sigma^-}\right)+0.5 \quad \text{(S18)}$$

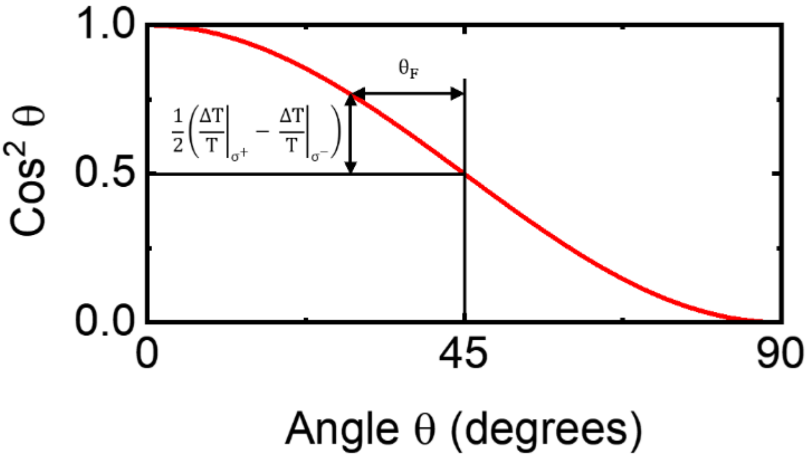

Fig. S7: Relation between $\Delta T/T$ and θ_F.

1 Jadidi, M. M. et al. Nonlinear terahertz absorption of graphene plasmons. *Nano Lett.* **16**, 2734-2738 (2016).

2 Jadidi, M. M. et al. Optical control of plasmonic hot carriers in graphene. *ACS Photonics* **6**, 302-307 (2019).

3 Yan, H. et al. Infrared spectroscopy of tunable Dirac terahertz magneto-plasmons in graphene. *Nano Lett.* **12**, 3766-3771 (2012).

4 Yan, H. et al. Tunable infrared plasmonic devices using graphene/insulator stacks. *Nat. Nanotechnol.* **7**, 330-334 (2012).

5 Han, J. W. et al. Plasmonic Terahertz Nonlinearity in Graphene Disks. *Adv. Photonics Res.* **3**, 2100218 (2022).
