# Supplementary material for: Strong transient magnetic fields induced by THz-driven plasmons in graphene disks
Source: Nat Commun. 2023 Nov 18;14:7493. doi: 10.1038/s41467-023-43412-x (PMC10657377; doi:10.1038/s41467-023-43412-x)
Supplement: Supplementary file 3 — Description of Additional Supplementary Files [file 41467_2023_43412_MOESM3_ESM.pdf]

Title: Supplementary Movie 1

Description: Strong transient magnetic fields induced by THz driven plasmons in graphene disks

The video file shows the spatio-temporal evolution of the magnetic field in the vicinity of the graphene disks after excitation with circularly polarized radiation. The strength of the magnetic field in the plane of the graphene disk is color coded, the arrows indicate the strength and direction of the magnetic field around the disk.
